# The Effects of Ursodeoxycholic Acid Pretreatment in an Experimental Setting of Extended Hepatectomy: A Feasibility Study

**DOI:** 10.7759/cureus.12120

**Published:** 2020-12-17

**Authors:** Anna Paspala, Dimitrios Papakonstantinou, Anastasia Prodromidou, Nick Danias, Anastasios Machairas, Georgios Agrogiannis, Nikolaos Machairas, Nikolaos J Zavras, Paulos Patapis, Emmanouil Pikoulis

**Affiliations:** 1 Third Department of Surgery, Attikon University General Hospital, School of Medicine, National and Kapodistrian University of Athens, Athens, GRC; 2 Obstetrics and Gynecology, Alexandra Hospital, National and Kapodistrian University of Athens, Athens, GRC; 3 Fourth Department of Surgery, Attikon University General Hospital, School of Medicine, National and Kapodistrian University of Athens, Athens, GRC; 4 First Department of Pathology, National and Kapodistrian University of Athens, Faculty of Medicine, Athens, GRC

**Keywords:** ursodeoxycholic acid, extended hepatectomy, liver resection, liver regeneration

## Abstract

Introduction

Liver regeneration is an exceptionally complex process, orchestrated by a multitude of growth factors and cytokines. Tumor necrosis factor-alpha (TNF-a) and interleukin-6 (Il-6) have a pivotal role in the initiation of the regenerative response. Ursodeoxycholic acid (UDCA) exhibits a liver protective effect that enhances liver growth after injury. The aim of the present study is to evaluate the effect of UDCA in the circulating levels of TNF-a and Il-6 in rats undergoing extended 80% hepatectomy.

Materials and methods

Twenty-two male Sprague Dawley rats were randomly assigned in an experimental (UDCA group) and a control group. Mice in the UDCA-group received oral pretreatment of UDCA for two weeks preoperatively at a dosage of 25 mg/kg/day. An 80% hepatic resection was performed in both groups by resecting the middle, inferior right, and left lateral liver lobes. The experiment ended 48 hours postoperatively.

Results

UDCA pretreatment significantly depressed circulating levels of both TNF-a and Il-6 after the conclusion of the experiment as compared to the control group (p=0.001 and p=0.01, respectively). Furthermore, TNF-a levels were significantly reduced before the institution of liver injury (p=0.02). Mice in the UDCA-group exhibited better liver growth as demonstrated by significantly increased Ki-67 and mitotic rate (p=0.04 and p=0.02, respectively). Finally, the liver regeneration rate (LRR) was significantly elevated in the experimental group (UDCA group, 54.5% vs control group, 35.8%; p=0.002) signifying enhanced liver growth kinetics.

Conclusion

UDCA reduces the expression of TNF-a and Il-6 during the priming phase of liver regeneration. An 80% hepatectomy model of acute liver failure exhibited enhanced liver regeneration in the experimental group, plausibly due to the immunomodulatory effects of UDCA.

## Introduction

It is well recognized that the liver has the extraordinary capacity to regenerate and regain its former functional capabilities even after extensive resection or parenchymal injury [1]. The extent of the regenerative process depends on the size of the underlying amount of injured or resected tissue, hence, an experimental model of 2/3 partial hepatectomy (PH) elicits a well-orchestrated process of liver cell proliferation, modulated by a wide variety of cytokines and growth factors [1,2].

Interleukin-6 (Il-6) is implicated to play a crucial role in this process through activation of the STAT3 and C/EBPβ/nuclear factor-IL6 transcription factors that eventually trigger the transition from the G0 to the G1 cell cycle phase [3,4]. Following PH, Il-6 is produced mainly by the hepatocytes in the remnant liver and to a lesser extent by nearby immune cells, in a regulated process that involves an interplay mechanism with hepatocyte growth factor (HGF) [5,6]. Similarly, tumor necrosis factor-alpha (TNF-a) has previously been shown to be an important initiator of liver regeneration in animal models [7,8]. Interaction of the tumor necrosis factor with its receptor (TNFR-1) creates a priming stimulus that induces hepatocellular regeneration through a molecular pathway akin to the one utilized by Il-6 [9].

Bile acids have been noted to enhance liver regeneration in mice animal models exhibiting a marked increase in cellular mitotic indices and hepatomegaly [10]. Ursodeoxycholic acid (UDCA) in particular, has been shown to improve hepatocellular proliferation, possibly decrease the effect of oxidative stress after PH [11], and moreover, protects against the deleterious effects of extracellular matrix metalloproteinases by upregulation of TIMP-1 [12]. UDCA has been demonstrated to decrease TNF-a and Il-6 expression in various tissues by dampening the elicited inflammatory immune response, however, it is uncertain whether this regulatory effect takes place after PH [13,14].

Following on from the previous report by Uzun et al. demonstrating the effectiveness of UDCA in a non-alcoholic fatty liver disease model, the aim of the present study is to further evaluate whether oral pretreatment with UDCA impacts the expression of TNF-a and Il-6 after extended hepatectomy and elucidate the potential role of UDCA in enhancing the liver regenerative process by regulating the post hepatectomy immune response [11].

## Materials and methods

Animals and groups

Based on the previous report by Uzun et al., the number of total samples required to attain 90% statistical power to detect a difference between experimental and control groups was determined to be n=22 [11]. For this purpose, 22 male, adult, non-pregnant Sprague Dawley rats were used in this study. The experiments were conducted in the Laboratory of Experimental Surgery “NS Christeas,” National and Kapodistrian University of Athens, with the approval of the local ethics committee. The rats were born in the approved, according to the directive 200/63/EU, animal facilities of the Laboratory of Experimental Surgery and Surgical Research “NS Christeas.”

In the current trial, all animals were four months old at the time of the procedure, weighing an average of 268 grams (range, 251-302 grams), and were individually housed in a temperature-controlled environment at 19±1°C with 12 hours light/dark cycles (light cycle from 08:00 AM to 08:00 PM). All rats were fed standard laboratory chow, with feed and water provided ad libitum.

Study design

The practice of the 3Rs (replacement, refinement, reduction) was adhered to. The animals were preoperatively randomized and divided into two groups as follows: the control group included 11 rats that underwent extended hepatectomy without any oral preparation, while the intervention group included 11 rats which were administered daily doses of UDCA, via the oral route, prior to the procedure. The UDCA solution was prepared by dissolving a 250 mg capsule (Ursofalk, Galenica SA, Athens, Greece) in 50 mL of normal saline. The 12 rats which comprised the intervention group were orally administered UDCA dissolved in saline via feeding tube, twice daily, at a dosage of 25 mg/kg/day for 14 days. The remaining ten rats comprising the control group were given saline in the same fashion as the intervention group. Blood samples were collected immediately before surgery and at the end of the experiment. Liver tissue samples were collected after animal euthanasia.

Surgery

The surgical procedures were performed under sterile conditions between 10:00 AM and 1:00 PM. Each rat was fasted for 12 hours preoperatively and was anesthetized by mask inhalation of diethyl ether. Maintenance anesthesia was achieved via continuous inhalation of the same agent. Preoperatively, the abdominal skin was shaved, and antisepsis was obtained with a 10% povidone-iodine solution. The abdomen was opened through a 3-4 cm midline laparotomy incision and after sufficient liver mobilization, 80% hepatectomy was achieved by resecting the middle, inferior right, and left lateral lobes as has been previously described by Martins et al. [15]. The choice of operation was made on the basis of creating a setting of acute liver failure for the present experiment. At the end of the operation, 10 mL of normal saline solution was injected into the peritoneal cavity to maintain hydration. All animal procedures were performed under the European Communities Council Directive of September 22, 2010 (276/33/20.10.2010) and approved by the competent Veterinary Directorate of Athens Prefecture, Greece (approval No.: 908/23.02.2016). All rats were fed a standard laboratory diet postoperatively and received analgesia with the use of subcutaneous buprenorphine (0.05 mg/kg) administered twice daily. No adverse events were encountered in the postoperative period. The animals were euthanized after 48 hours postoperatively by exsanguination after deep sedation was achieved with ether inhalation.

Liver regeneration rate

The liver regeneration rate (LRR) was calculated after the end of the experiment using the formula derived by Okano et al. [16,17]:

Liver regeneration rate (%) = 100 x [C - (A - B)] / A

In this equation, A stands for the estimated whole liver weight at surgery, B is the wet tissue weight of the excised liver, and C is the weight of the remnant liver at the time of sacrifice. The whole liver weight (A) was estimated by sacrificing five rats of the same age not included in the control or intervention groups, weighing an average of 270 grams. The whole liver weight at the time of operation was estimated to be on average 11.59 grams.

Serum TNF-a and IL-6 values and immunohistochemical analysis

The serum levels of these proinflammatory cytokines in each rat were measured by enzyme-linked immunosorbent assay (ELISA) using commercially available kits (TNF-a mouse ELISA Kit, Cayman, Michigan, USA, and Il-6 mouse ELISA Kit, Cayman, Michigan, USA) and expressed as picograms per milliliter. The ELISAs on blood samples were performed exactly before the extended hepatectomy and 48 hours postoperatively. Slides of 5-6 μm thick of each regenerating remnant liver specimen were deparaffinized. These were stained with hematoxylin and eosin (H&E) to evaluate the mitotic figure count while immunohistochemistry was used to evaluate Ki67 antigen.

Statistical analysis

The assessment of the normality of data distribution was performed with the Shapiro-Wilk test. Comparisons between two groups with quantitative data were made using the Student's t-test when data were normally distributed with equal variances, the Welch's t-test when data were normally distributed with unequal variances, and the Mann-Whitney U test when data did not follow a normal distribution. All the tests were two-tailed. Results were considered statistically significant if the p-value was less than 0.05. 

## Results

The mean body weight of the animals preoperatively was 261.33±13.64 grams and 254.83±14.98 grams in the control and the UDCA groups, respectively (p=0.22). The mean weight of extended hepatectomy specimen was 6.79±0.95 grams, in the control group, whereas, in the UDCA group, the mean weight of extended hepatectomy specimen was 6.59±0.73 grams (p=0.57). Additionally, the mean weight of the remnant liver was 4.64±0.74 grams and 5.24±0.64 grams in the control group and UDCA group, respectively (Table 1).

**Table 1:** Comparative values between the experimental and control groups. SD: standard deviation, LRR: liver regeneration rate; UDCA: ursodeoxycholic acid; TNF-a: tumor necrosis factor-alpha; Il-6: interleukin-6

	Control group (n=11, mean±SD)	UDCA group (n=11, mean±SD)	p-Value
Animal weight	261.33±13.64 g	254.83±14.98 g	0.22
Weight of resected liver	6.79±0.95 g	6.59±0.73 g	0.57
Weight of the remnant liver	4.64±0.74 g	5.24±0.64 g	0.053
TNF-a (preoperative)	46.57±22.57 pg/mL	29.44±11.51 pg/mL	0.02
TNF-a (postoperative)	75.21±32.8 pg/mL	36.75±8.68 pg/mL	0.001
Il-6 (preoperative)	18.96±10.45 pg/mL	12.50±8.25 pg/mL	0.1
Il-6 (postoperative)	32.95±13.17 pg/mL	18.98±9.5 pg/mL	0.01
LRR (%)	35.81±12.49	54.92±14.37	0.002

Biochemical analysis

Mean TNF-a levels were found to be significantly elevated postoperatively in both control and UDCA groups (control group; 46.57±22.57 pg/mL preoperatively vs 75.21±32.8 pg/mL postoperatively, p=0.02. UDCA group; 29.44±11.51 pg/mL preoperatively versus 36.75±8.68 pg/mL postoperatively, p= 0.03).

**Figure 1:**
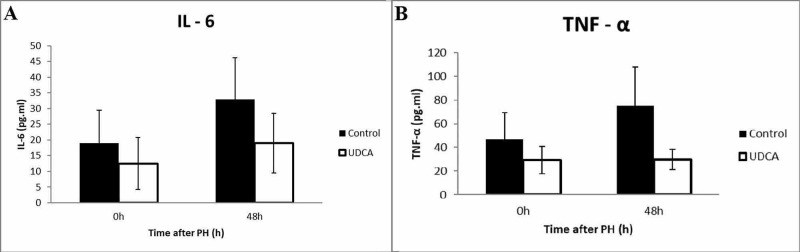
Comparison between serum levels of Il-6 (A) and TNF-a (B) during pre and postoperative sampling. TNF-a: tumor necrosis factor-alpha; Il-6: interleukin-6

When the two groups were compared, a significant increase of TNF-a levels was observed in the control group in comparison to the UDCA group both preoperatively (46.57±22.57 vs 29.44±11.51 pg/mL, p=0.02) as well as postoperatively (75.21±32.8 vs 36.75±8.68 pg/mL, p=0.001, Figure 1A).

Concerning Il-6 level measurements, a statistically significant increase was noted in the postoperative period in the control group (18.96±10.45 pg/mL preoperatively vs 32.95±13.17 pg/mL postoperatively, p=0.01). In the UDCA group, no statistically significant difference existed between pre and postoperative values (12.50±8.25 pg/mL preoperatively vs 18.98±9.5 pg/mL postoperatively, p=0.08). An intergroup comparison in regards to Il-6 levels revealed no difference in preoperative Il-6 values between control and UDCA groups (18.96±10.45 pg/mL vs 12.50±8.25 pg/mL, respectively, p=0.1), however, a significant reduction of the postoperative Il-6 levels in the UDCA group was noted when compared to the control group (32.95±13.17 pg/mL vs 18.98±9.5 pg/mL, respectively p=0.01, Figure 1B).

Histological examination

**Figure 2:**
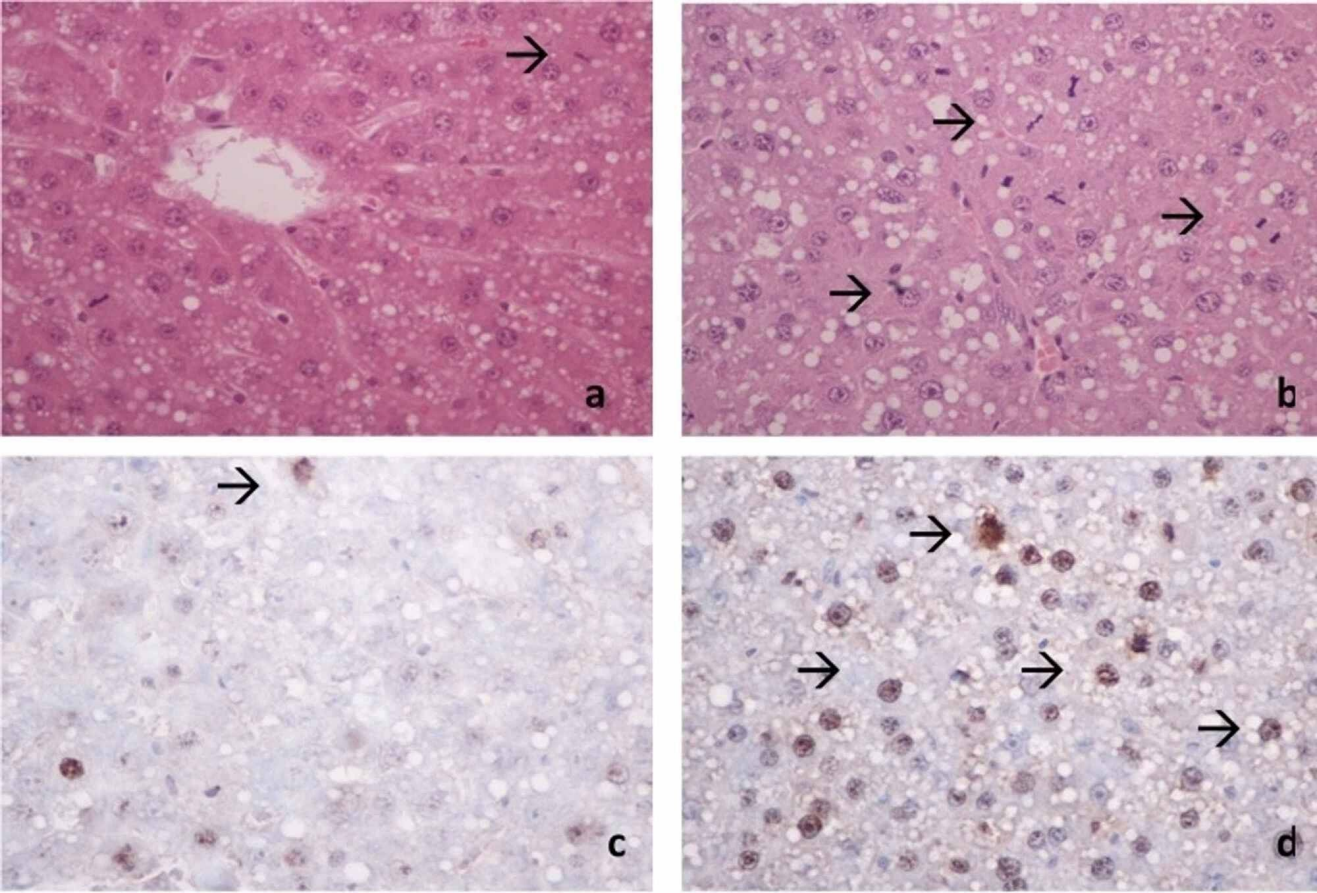
Mitotic rates in the control group (A) and UDCA group (B). Representative image of Ki-67 antigen immunohistochemistry in the control group (C) vs the UDCA group (D).

Visual histopathologic examination of liver tissue (Figure 2) revealed significantly elevated mitotic figures in the UDCA group in comparison to the control group (43±11.42 vs 27.83±18.673, p=0.02). Moreover, the Ki-67-positive nuclei count measured in ten optic fields was evaluated in both groups revealing a statistically significant difference in the UDCA group compared with the control group (14.75±7.8 vs 8.1±4.1, p=0.04).

Liver regeneration rate

**Figure 3:**
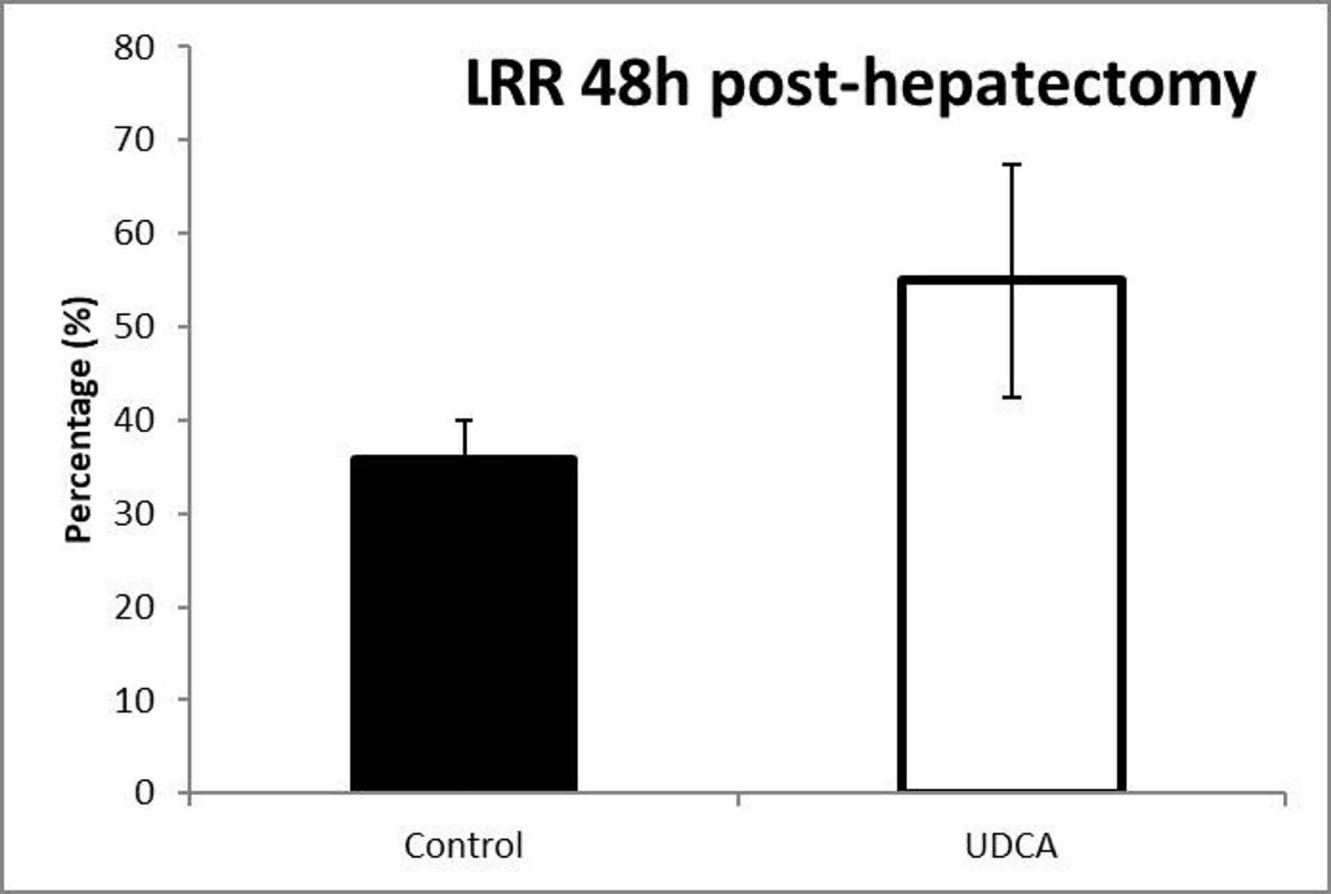
Postoperative liver growth kinetics.

The mean values of LRR were 54.92%±14.37% in the UDCA group compared to 35.81%±12.49% in the control group. The comparison of the values of LRR between the control group and the UDCA group revealed a statistically significant increase of LRR in the UDCA group after 80% hepatectomy (p=0.002) (Figure 3).

## Discussion

Despite recent advances in the field, hepatic regeneration remains one of the most complex phenomena encountered in physiology. A growing body of evidence suggests that this process can in fact be ameliorated by extrinsic supplementation of bile salts and specifically UDCA, through a poorly understood mechanism. Ishizaki et al. utilized a model of chemically induced liver injury by injecting concanavalin A to investigate the protective effect of UDCA pretreatment immediately before the injurious insult [18]. His results emphatically demonstrated that UDCA suppresses the overall immune response by lowering the levels of TNF-a, Il-6, and macrophage inflammatory protein-2 (MIP-2). Buryova et al. further expanded on these results by demonstrating that oral pretreatment with UDCA significantly attenuated liver damage after eight days following bile duct ligation [12]. Furthermore, they observed increased retention of intracellular glycogen in hepatocytes and probable inhibition of ADAM17, a molecule theorized to partake in the regulation of cytokine uptake during the priming phase of hepatic regeneration. The study by Uzun et al. is the third documenting the hepatoprotective effects of UDCA [11]. In a model of induced non-alcoholic fatty hepatitis, they eloquently demonstrated that UDCA pretreatment leads to higher rates of liver regeneration through a mechanism that reduces intracellular oxidative stress.

Based on these three pivotal studies, it becomes apparent that UDCA exerts its effects through at least two main mechanisms; firstly by modulation of the priming inflammatory process in the early phase of liver regeneration and secondly by establishing a cytoprotective effect in the regenerating liver microenvironment during the later phase. In regards to the former mechanism, however, a contradiction arises. On one hand, TNF-a and Il-6 are regarded as key players in the initiation of the regenerative process after hepatectomy, based on the results of studies on knock-out animals [9,19,20], and on the other hand, UDCA seems to decrease circulating levels of both cytokines in hepatic regeneration after chemically induced injury [12,18]. In the present study, we endeavored to evaluate whether the immunomodulatory effects of UDCA are preserved in hepatic resection models in an effort to determine whether a different regenerative process is implicated as opposed to the one evident after chemical injury.

Oral administration of UDCA has previously been shown to be effective in establishing adequate intracellular levels [11,18,21]. The choice of dosage was selected based on previous reports to ensure reproducibility, while the duration of UDCA pretreatment in the experimental group was two weeks to ensure adequate corporeal distribution of the drug in all participating animals [11]. A model of 80% extended hepatectomy was chosen instead of traditional 2/3 PH models with the rationale of maximizing the elicited systematic and local inflammatory response while ensuring the survivability of the operated mice. In fact, no animal died before the conclusion of the presently described experimental process. As pointed out in the study by Trautwein et al. regarding the kinetics of TNF-a and Il-6 after PH, serum levels for both cytokines peak in two to four hours after surgery and gradually return to a new baseline in 24-48 hours [22]. Consequently, we selected to end the experiment in 48 hours postoperatively so as to ensure that measurements are not made during the acute-phase response but still remain within the early phase of hepatic regeneration.

The obtained results imply that the hepatoprotective effect of UDCA is maintained after hepatic resection as was demonstrated by the higher rates of Ki-67 and mitotic counts in the experimental group. Taking into account that UDCA is a hydrophilic conjugated bile salt, its effects on enhancing cellular proliferation are indirect and cannot be explained by nuclear receptor binding and activation. The results published by Barone et al. serve to validate this hypothesis [23]. In their study, the in vivo beneficial effects of UDCA could not be replicated in isolated hepatocellular cell cultures. Castro et al. postulated that a possible interaction with microRNA could explain this phenomenon [24]. By employing a 70% extended hepatectomy model they observed significant induction of miR-21 levels by oral UDCA pretreatment, suggesting that this molecule may be the effector of cell cycle progression underlying the pro-proliferative capacity of UDCA.

Concerning the serum levels of TNF-a and Il-6, our results indicate that the circulation of both cytokines was significantly reduced in mice in the experimental group (Figure 1). This finding verifies the modulating effects of UDCA in the model of extended hepatectomy in the same way as previously reported in models of chemical injury [12,18]. In addition, the rate of TNF-a and Il-6 increase following extended hepatectomy was markedly increased in the control group (TNF-a; 61.4% increase after surgery, Il-6; 73.7% increase) relatively to the UDCA group (TNF-a; 19.8% increase after surgery, Il-6; 51.8% increase). Interestingly, preoperative blood samples revealed that pretreatment suppressed TNF-a (but not Il-6) levels to a statistically significant degree, even without the addition of liver injury. This denotes that UDCA may exert its immunomodulatory role even in quiescent macrophages.

The LRR is a useful adjunct for estimating the kinetics of liver growth, first described by Okano et al. [16]. Although there were no calculable differences in the weights of resected and remnant livers (Table 1), the rate of growth of LRR demonstrated a significant increase in the group of mice that received oral UDCA supplementation before surgery. This further solidifies the regenerative capacity of UDCA both microscopically and macroscopically.

The present study is the first attempt at using a model of 80% extended hepatectomy that allows assessment of the modulatory impact of UDCA on the main cytokines implicated in the initiation of liver regeneration, in a setting of acute liver failure. Our results demonstrate this experimental model is feasible and reproducible with observations that are on par with the growing body of literature on the topic. Moreover, they verify the hypothesis that the main modulatory mechanisms of action of UDCA are the same in the acute liver failure setting following extensive hepatectomy. Nevertheless, there are important limitations that should be noted. First of all, although the study was adequately powered, based on existing literature, to detect statistically significant differences between the two groups, the overall magnitude of the observed differences may have been underestimated due to the relatively small sample size. Additionally, although the present study indicates that UDCA exerts its beneficial effects even in cases of acute liver failure, it is still uncertain to what extent this is through direct action on intracellular molecular targets or whether it is through indirect action through the immune system tweaking.

## Conclusions

In conclusion, the current feasibility study demonstrated that UDCA reduced the expression of TNF-a and Il-6 during the priming phase of liver regeneration, in a setting of acute liver failure. Furthermore, it sets the basis for creating a research model for evaluating the effector molecules of the UDCA mediated enhanced hepatocellular proliferation in cases of pronounced generalized and localized physiological stress.
